# Tetra­aqua­(2,2′-diamino-4,4′-bi-1,3-thia­zole-κ^2^ *N* ^3^,*N* ^3′^)nickel(II) bis­(pyridine-2,6-dicarboxyl­ato-κ^3^ *O* ^2^,*N*,*O* ^6^)nickel(II) trihydrate

**DOI:** 10.1107/S1600536809004589

**Published:** 2009-02-18

**Authors:** Bing-Xin Liu, Yan-Ping Yu, Zen Cao, Liang-Jun Zhang

**Affiliations:** a Department of Chemistry, Shanghai University, Shanghai 200444, People’s Republic of China; b Department of Petroleum and Chemical Industry, Guangxi Vocational and Technical Institute of Industry, People’s Republic of China

## Abstract

The crystal structure of the title compound, [Ni(C_6_H_6_N_4_S_2_)(H_2_O)_4_][Ni(C_7_H_3_NO_4_)_2_]·3H_2_O, consists of Ni^II^ complex cations, Ni^II^ complex anions and lattice water mol­ecules. The Ni^II^ ions in both the complex cation and anion assume a distorted octa­hedral coordination geometry. O—H⋯O, N—H⋯O and C—H⋯S hydrogen bonds occur in the crystal structure.

## Related literature

For general background, see: Waring (1981 ▶); Fisher *et al.* (1985 ▶). For a related structure, see: Liu *et al.* (2003 ▶); Zhang *et al.* (2006 ▶). For synthesis, see: Erlenmeyer (1948 ▶).
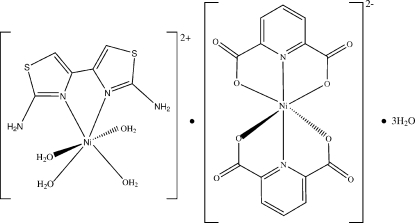


## Experimental

### 

#### Crystal data

[Ni(C_6_H_6_N_4_S_2_)(H_2_O)_4_][Ni(C_7_H_3_NO_4_)_2_]·3H_2_O
*M* *_r_* = 772.01
Triclinic, 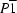

*a* = 11.4756 (13) Å
*b* = 11.5609 (13) Å
*c* = 13.2667 (15) Å
α = 65.3590 (10)°
β = 82.1140 (11)°
γ = 66.0180 (10)°
*V* = 1460.6 (3) Å^3^
*Z* = 2
Mo *K*α radiation
μ = 1.51 mm^−1^
*T* = 295 K
0.23 × 0.18 × 0.15 mm

#### Data collection

Bruker APEX CCD diffractometer
Absorption correction: multi-scan (*SADABS*; Sheldrick, 1996 ▶) *T*_min_ = 0.710, *T*_max_ = 0.795
7580 measured reflections
5052 independent reflections
4181 reflections with *I* > 2σ(*I*)
*R*_int_ = 0.018

#### Refinement

*R*[*F* ^2^ > 2σ(*F* ^2^)] = 0.034
*wR*(*F* ^2^) = 0.084
*S* = 1.05
5052 reflections
412 parameters
H-atom parameters constrained
Δρ_max_ = 0.31 e Å^−3^
Δρ_min_ = −0.43 e Å^−3^

### 

Data collection: *SMART* (Bruker, 2004 ▶); cell refinement: *SAINT* (Bruker, 2004 ▶); data reduction: *SAINT*; program(s) used to solve structure: *SIR92* (Altomare *et al.*, 1993 ▶); program(s) used to refine structure: *SHELXL97* (Sheldrick, 2008 ▶); molecular graphics: *ORTEP-3 for Windows* (Farrugia, 1997 ▶); software used to prepare material for publication: *WinGX* (Farrugia, 1999 ▶).

## Supplementary Material

Crystal structure: contains datablocks I, global. DOI: 10.1107/S1600536809004589/xu2461sup1.cif

Structure factors: contains datablocks I. DOI: 10.1107/S1600536809004589/xu2461Isup2.hkl

Additional supplementary materials:  crystallographic information; 3D view; checkCIF report

## Figures and Tables

**Table 1:** Hydrogen-bond geometry (Å, °)

*D*—H⋯*A*	*D*—H	H⋯*A*	*D*⋯*A*	*D*—H⋯*A*
O1—H1*A*⋯O14^i^	0.86	1.75	2.606 (3)	174
O1—H1*B*⋯O21^ii^	0.82	1.92	2.747 (3)	176
O2—H2*A*⋯O12^iii^	0.77	2.01	2.772 (3)	169
O2—H2*B*⋯O22^ii^	0.76	2.12	2.874 (3)	170
O3—H3*A*⋯O3*W*^ii^	0.78	1.98	2.742 (3)	169
O3—H3*B*⋯O11^iii^	0.75	1.94	2.683 (3)	173
O4—H4*A*⋯O24^iv^	0.83	1.89	2.717 (4)	179
O4—H4*B*⋯O1*W*	0.74	2.16	2.820 (4)	149
O1*W*—H1*WA*⋯O23^iv^	0.91	2.01	2.907 (4)	166
O1*W*—H1*WB*⋯O24^iii^	0.75	2.41	3.150 (5)	169
O2*W*—H2*WB*⋯O3*W*^v^	0.72	2.41	3.104 (4)	162
O2*W*—H2*WA*⋯O14	0.79	2.02	2.815 (4)	174
O3*W*—H3*WA*⋯O22^vi^	0.73	2.24	2.934 (3)	158
O3*W*—H3*WB*⋯O21	0.88	2.10	2.892 (3)	149
N32—H32*A*⋯O12	0.98	1.97	2.940 (5)	169
N32—H32*B*⋯O2	0.95	2.19	2.996 (5)	142
N34—H34*A*⋯O2*W*^vii^	0.92	1.98	2.886 (4)	168
N34—H34*B*⋯O3	0.92	2.12	2.952 (4)	149
C12—H12⋯S31	0.93	2.71	3.631 (4)	170

Symmetry codes: (i) 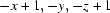; (ii) 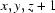; (iii) 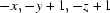; (iv) 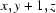; (v) 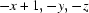; (vi) 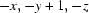; (vii) 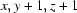.

**Fig. 1.**
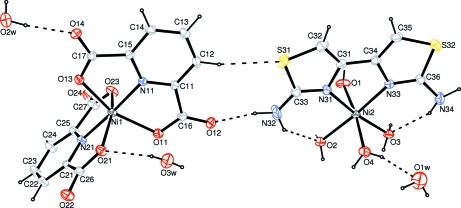
The molecular structure of (I) with 30% probability displacement ellipsoids (arbitrary spheres for H atoms), dashed lines showing the hydrogen bonding.

## supplementary crystallographic information

### Comment

Transition metal complexes of 2,2'-diamino-4,4'-bi-1,3-thiazole (DABT) have shown potential application in some fields, for example, a Co^II^ complex and a Ni^II^ complex with the DABT ligand have been found to be effective inhibitors of DNA synthesis of tumor cell (Waring, 1981; Fisher *et al.*, 1985). As part of serial structural investigation of metal complexes with DABT, the title Ni^II^ complex was prepared in the laboratory and its X-ray structure is presented here.

The crystal of title compound consists of Ni^II^ complex cations, Ni^II^ complex anions and lattice water molecules (Fig. 1). Within the complex cation, the Ni^II^ ion is coordinated by a DABT ligand and four water molecules in a distorted octahedral geometry. The thiazole rings of DABT are approximately coplanar with the dihedral angle of 6.2 (2)° between thiazole rings. The average of Ni—N bond distance of 2.082 (2) Å is comparable to the Ni—N bond distance of 2.103 (4) Å found in [Ni(C_6_H_6_N_4_S_2_)_2_(H_2_O)_2_][Ni(C_7_H_3_NO_4_)_2_].5(H_2_O) (Zhang *et al.*, 2006) and 2.113 (2) Å found in [Ni(C_8_H_4_O_4_)(C_6_H_6_N_4_S_2_)_2_].3.5H_2_O (Liu *et al.*, 2003).

In the complex anion, the Ni^II^ ion is chelated by two pyridinedicarboxylate (pdc) dianions with a distorted octahedral geometry. Two planar pdc ligands are nearly perpendicular to each other, the dihedral angle being 88.46 (8)°.

The extensive hydrogen bonding between lattice water molecules, complex cainos and complex anions helps to stabilize the crystal structure (Table 1).

### Experimental

The microcrystals of DABT were obtained in the manner reported by Erlenmeyer (1948). An aqueous solution (20 ml) containing DABT (0.20 g, 1 mmol) and NiCl_2_ (0.13 g, 1 mmol) was mixed with another aqueous solution (10 ml) of 2,6-pyridinedicarboxylic acid (0.17 g, 1 mmol) and NaOH (0.08 g, 2 mmol). The mixture was refluxed for 6 h. The solution was filtered after cooling to room temperature. Green single crystals were obtained from the filtrate after 30 d.

### Refinement

Aromatic H atoms were placed in calculated positions with C—H = 0.93 Å and were included in the final cycles of refinement in riding mode with *U*_iso_(H) = 1.2*U*_eq_(C). H atoms on N atom of amino group of DABT and on O atom of water molecules were located in a difference Fourier map and also included in the final cycles of refinement in riding mode with *U*_iso_(H) = 1.5*U*_eq_(O) or 1.2*U*_eq_(N).

### Crystal data

**Table:**

[Ni(C_6_H_6_N_4_S_2_)(H_2_O)_4_][Ni(C_7_H_3_NO_4_)_2_]·3H_2_O	*Z* = 2
*M**_r_* = 772.01	*F*(000) = 792
Triclinic, *P*1	*D*_x_ = 1.755 Mg m^−^^3^
Hall symbol: -P 1	Mo *K*α radiation, λ = 0.71073 Å
*a* = 11.4756 (13) Å	Cell parameters from 4980 reflections
*b* = 11.5609 (13) Å	θ = 2.0–25.0°
*c* = 13.2667 (15) Å	µ = 1.51 mm^−^^1^
α = 65.359 (1)°	*T* = 295 K
β = 82.1140 (11)°	Prism, green
γ = 66.018 (1)°	0.23 × 0.18 × 0.15 mm
*V* = 1460.6 (3) Å^3^	

### Data collection

**Table:**

Bruker APEX CCD diffractometer	5052 independent reflections
Radiation source: fine-focus sealed tube	4181 reflections with *I* > 2σ(*I*)
graphite	*R*_int_ = 0.018
φ and ω scans	θ_max_ = 25.0°, θ_min_ = 2.1°
Absorption correction: multi-scan (*SADABS*; Sheldrick, 1996)	*h* = −13→7
*T*_min_ = 0.710, *T*_max_ = 0.795	*k* = −13→13
7580 measured reflections	*l* = −15→15

### Refinement

**Table:**

Refinement on *F*^2^	Primary atom site location: structure-invariant direct methods
Least-squares matrix: full	Secondary atom site location: difference Fourier map
*R*[*F*^2^ > 2σ(*F*^2^)] = 0.034	Hydrogen site location: inferred from neighbouring sites
*wR*(*F*^2^) = 0.084	H-atom parameters constrained
*S* = 1.05	*w* = 1/[σ^2^(*F*_o_^2^) + (0.0368*P*)^2^ + 0.7271*P*] where *P* = (*F*_o_^2^ + 2*F*_c_^2^)/3
5052 reflections	(Δ/σ)_max_ = 0.001
412 parameters	Δρ_max_ = 0.31 e Å^−^^3^
0 restraints	Δρ_min_ = −0.43 e Å^−^^3^

### Special details

**Table:**

Geometry. All e.s.d.'s (except the e.s.d. in the dihedral angle between two l.s. planes) are estimated using the full covariance matrix. The cell e.s.d.'s are taken into account individually in the estimation of e.s.d.'s in distances, angles and torsion angles; correlations between e.s.d.'s in cell parameters are only used when they are defined by crystal symmetry. An approximate (isotropic) treatment of cell e.s.d.'s is used for estimating e.s.d.'s involving l.s. planes.
Refinement. Refinement of *F*^2^ against ALL reflections. The weighted *R*-factor *wR* and goodness of fit *S* are based on *F*^2^, conventional *R*-factors *R* are based on *F*, with *F* set to zero for negative *F*^2^. The threshold expression of *F*^2^ > σ(*F*^2^) is used only for calculating *R*-factors(gt) *etc*. and is not relevant to the choice of reflections for refinement. *R*-factors based on *F*^2^ are statistically about twice as large as those based on *F*, and *R*- factors based on ALL data will be even larger.

### Fractional atomic coordinates and isotropic or equivalent isotropic displacement parameters (Å^2^)

**Table:**

	*x*	*y*	*z*	*U*_iso_*/*U*_eq_	
Ni1	0.16770 (3)	0.07786 (4)	0.17826 (3)	0.02475 (11)	
Ni2	0.23271 (3)	0.51033 (4)	0.71455 (3)	0.02385 (11)	
O1	0.2614 (2)	0.3223 (2)	0.84334 (17)	0.0390 (6)	
H1A	0.3396	0.2657	0.8611	0.059*	
H1B	0.2134	0.3025	0.8940	0.059*	
O2	0.06240 (19)	0.4957 (2)	0.68468 (17)	0.0343 (5)	
H2A	−0.0007	0.5604	0.6691	0.051*	
H2B	0.0430	0.4451	0.7356	0.051*	
O3	0.13816 (18)	0.6134 (2)	0.81574 (16)	0.0319 (5)	
H3A	0.1392	0.5667	0.8783	0.048*	
H3B	0.0692	0.6533	0.7976	0.048*	
O4	0.1730 (2)	0.7000 (2)	0.58611 (17)	0.0414 (6)	
H4A	0.1238	0.7351	0.5318	0.062*	
H4B	0.1773	0.7579	0.5939	0.062*	
O11	0.10601 (19)	0.2234 (2)	0.25192 (17)	0.0337 (5)	
O12	0.1791 (2)	0.2960 (2)	0.35197 (18)	0.0389 (5)	
O13	0.29701 (19)	−0.0576 (2)	0.11358 (17)	0.0326 (5)	
O14	0.50368 (19)	−0.1545 (2)	0.08943 (17)	0.0349 (5)	
O21	0.10956 (19)	0.2430 (2)	0.01530 (16)	0.0311 (5)	
O22	−0.0369 (2)	0.3366 (2)	−0.12130 (17)	0.0382 (5)	
O23	0.1450 (2)	−0.0753 (2)	0.32817 (17)	0.0362 (5)	
O24	0.0132 (3)	−0.1844 (3)	0.4064 (2)	0.0612 (8)	
N11	0.3291 (2)	0.0700 (2)	0.21889 (19)	0.0232 (5)	
N21	0.0100 (2)	0.0718 (2)	0.14401 (19)	0.0239 (5)	
N31	0.3493 (2)	0.4052 (2)	0.62139 (19)	0.0290 (6)	
N32	0.2175 (3)	0.3633 (4)	0.5323 (3)	0.0644 (10)	
H32A	0.2169	0.3353	0.4722	0.077*	
H32B	0.1419	0.4222	0.5525	0.077*	
N33	0.4079 (2)	0.5113 (2)	0.74188 (19)	0.0257 (5)	
N34	0.3714 (3)	0.6488 (3)	0.8419 (2)	0.0467 (8)	
H34A	0.4167	0.6570	0.8887	0.056*	
H34B	0.2868	0.6592	0.8476	0.056*	
S31	0.47085 (9)	0.26783 (9)	0.50388 (7)	0.0432 (2)	
S32	0.61255 (8)	0.50673 (9)	0.80414 (8)	0.0412 (2)	
C11	0.3283 (3)	0.1372 (3)	0.2798 (2)	0.0248 (6)	
C12	0.4413 (3)	0.1217 (3)	0.3184 (3)	0.0343 (7)	
H12	0.4412	0.1698	0.3597	0.041*	
C13	0.5547 (3)	0.0337 (3)	0.2948 (3)	0.0360 (8)	
H13	0.6320	0.0210	0.3209	0.043*	
C14	0.5528 (3)	−0.0360 (3)	0.2316 (3)	0.0297 (7)	
H14	0.6284	−0.0953	0.2148	0.036*	
C15	0.4374 (3)	−0.0157 (3)	0.1945 (2)	0.0238 (6)	
C16	0.1940 (3)	0.2267 (3)	0.2975 (2)	0.0279 (7)	
C17	0.4124 (3)	−0.0821 (3)	0.1264 (2)	0.0263 (6)	
C21	−0.0484 (3)	0.1524 (3)	0.0445 (2)	0.0238 (6)	
C22	−0.1516 (3)	0.1387 (3)	0.0152 (2)	0.0316 (7)	
H22	−0.1932	0.1958	−0.0540	0.038*	
C23	−0.1914 (3)	0.0377 (4)	0.0917 (3)	0.0384 (8)	
H23	−0.2598	0.0254	0.0737	0.046*	
C24	−0.1295 (3)	−0.0449 (3)	0.1947 (3)	0.0356 (8)	
H24	−0.1557	−0.1127	0.2467	0.043*	
C25	−0.0284 (3)	−0.0246 (3)	0.2186 (2)	0.0279 (7)	
C26	0.0113 (3)	0.2535 (3)	−0.0281 (2)	0.0267 (7)	
C27	0.0489 (3)	−0.1018 (3)	0.3268 (3)	0.0361 (8)	
C31	0.4781 (3)	0.3740 (3)	0.6331 (2)	0.0270 (7)	
C32	0.5566 (3)	0.3003 (3)	0.5773 (3)	0.0382 (8)	
H32	0.6450	0.2712	0.5783	0.046*	
C33	0.3309 (3)	0.3552 (3)	0.5550 (3)	0.0364 (8)	
C34	0.5109 (3)	0.4281 (3)	0.7014 (2)	0.0281 (7)	
C35	0.6263 (3)	0.4128 (3)	0.7279 (3)	0.0372 (8)	
H35	0.7033	0.3589	0.7079	0.045*	
C36	0.4474 (3)	0.5613 (3)	0.7966 (3)	0.0307 (7)	
O1W	0.1949 (3)	0.9552 (3)	0.5228 (2)	0.0749 (9)	
H1WA	0.1810	0.9596	0.4548	0.112*	
H1WB	0.1411	1.0142	0.5309	0.112*	
O2W	0.5287 (2)	−0.3045 (3)	−0.0391 (2)	0.0537 (7)	
H2WB	0.5946	−0.3508	−0.0270	0.081*	
H2WA	0.5167	−0.2587	−0.0049	0.081*	
O3W	0.1763 (2)	0.4471 (2)	0.03785 (18)	0.0483 (6)	
H3WA	0.1475	0.4837	0.0745	0.072*	
H3WB	0.1492	0.3796	0.0574	0.072*	

### Atomic displacement parameters (Å^2^)

**Table:**

	*U*^11^	*U*^22^	*U*^33^	*U*^12^	*U*^13^	*U*^23^
Ni1	0.0183 (2)	0.0276 (2)	0.0284 (2)	−0.00704 (16)	−0.00210 (15)	−0.01206 (16)
Ni2	0.0214 (2)	0.0248 (2)	0.0232 (2)	−0.00605 (16)	−0.00041 (15)	−0.00997 (16)
O1	0.0254 (12)	0.0335 (12)	0.0358 (12)	−0.0041 (10)	0.0056 (10)	−0.0017 (10)
O2	0.0263 (11)	0.0338 (12)	0.0377 (12)	−0.0071 (10)	−0.0021 (9)	−0.0130 (10)
O3	0.0244 (11)	0.0359 (12)	0.0290 (11)	−0.0038 (9)	−0.0016 (9)	−0.0138 (9)
O4	0.0510 (15)	0.0320 (12)	0.0317 (12)	−0.0134 (11)	−0.0126 (11)	−0.0025 (10)
O11	0.0204 (11)	0.0401 (12)	0.0423 (13)	−0.0039 (10)	−0.0023 (10)	−0.0245 (10)
O12	0.0338 (13)	0.0422 (13)	0.0471 (14)	−0.0064 (10)	−0.0004 (10)	−0.0312 (11)
O13	0.0244 (12)	0.0385 (12)	0.0417 (13)	−0.0083 (10)	−0.0025 (9)	−0.0247 (10)
O14	0.0282 (12)	0.0351 (12)	0.0417 (13)	−0.0035 (10)	0.0006 (10)	−0.0239 (10)
O21	0.0299 (12)	0.0327 (11)	0.0303 (11)	−0.0160 (10)	−0.0016 (9)	−0.0077 (9)
O22	0.0388 (13)	0.0392 (12)	0.0274 (12)	−0.0171 (11)	−0.0044 (10)	−0.0013 (10)
O23	0.0287 (12)	0.0432 (13)	0.0308 (12)	−0.0153 (10)	−0.0064 (9)	−0.0060 (10)
O24	0.0633 (18)	0.0762 (19)	0.0344 (14)	−0.0482 (16)	−0.0134 (12)	0.0120 (13)
N11	0.0191 (12)	0.0251 (12)	0.0261 (13)	−0.0069 (10)	−0.0008 (10)	−0.0117 (10)
N21	0.0195 (12)	0.0266 (13)	0.0228 (13)	−0.0079 (10)	−0.0002 (10)	−0.0080 (10)
N31	0.0305 (14)	0.0314 (14)	0.0260 (13)	−0.0096 (12)	0.0009 (11)	−0.0147 (11)
N32	0.048 (2)	0.098 (3)	0.077 (2)	−0.0195 (19)	−0.0009 (18)	−0.070 (2)
N33	0.0242 (13)	0.0241 (12)	0.0262 (13)	−0.0080 (11)	0.0006 (10)	−0.0090 (10)
N34	0.0357 (16)	0.0591 (19)	0.067 (2)	−0.0165 (15)	0.0037 (15)	−0.0468 (17)
S31	0.0550 (6)	0.0434 (5)	0.0343 (5)	−0.0156 (4)	0.0099 (4)	−0.0242 (4)
S32	0.0301 (5)	0.0451 (5)	0.0524 (5)	−0.0159 (4)	−0.0051 (4)	−0.0198 (4)
C11	0.0259 (16)	0.0219 (14)	0.0279 (15)	−0.0099 (12)	−0.0002 (12)	−0.0103 (12)
C12	0.0332 (18)	0.0415 (18)	0.0411 (19)	−0.0174 (15)	−0.0003 (15)	−0.0250 (16)
C13	0.0225 (16)	0.0424 (19)	0.050 (2)	−0.0126 (15)	−0.0024 (14)	−0.0234 (16)
C14	0.0181 (15)	0.0298 (16)	0.0389 (18)	−0.0074 (13)	0.0012 (13)	−0.0136 (14)
C15	0.0224 (15)	0.0229 (14)	0.0261 (15)	−0.0097 (12)	0.0024 (12)	−0.0092 (12)
C16	0.0262 (16)	0.0251 (15)	0.0264 (16)	−0.0051 (13)	−0.0019 (13)	−0.0088 (13)
C17	0.0281 (17)	0.0217 (15)	0.0257 (16)	−0.0074 (13)	−0.0010 (13)	−0.0079 (12)
C21	0.0186 (15)	0.0260 (15)	0.0254 (15)	−0.0061 (12)	−0.0004 (12)	−0.0111 (12)
C22	0.0252 (16)	0.0406 (18)	0.0270 (16)	−0.0118 (14)	−0.0045 (13)	−0.0107 (14)
C23	0.0301 (18)	0.057 (2)	0.0347 (18)	−0.0252 (17)	−0.0010 (14)	−0.0162 (16)
C24	0.0324 (18)	0.0446 (19)	0.0312 (17)	−0.0242 (16)	0.0031 (14)	−0.0081 (15)
C25	0.0269 (16)	0.0309 (16)	0.0237 (15)	−0.0112 (13)	−0.0014 (13)	−0.0081 (13)
C26	0.0226 (16)	0.0248 (15)	0.0304 (17)	−0.0061 (13)	0.0004 (13)	−0.0116 (13)
C27	0.0306 (18)	0.0411 (19)	0.0297 (18)	−0.0144 (15)	−0.0036 (14)	−0.0058 (15)
C31	0.0276 (16)	0.0228 (15)	0.0264 (16)	−0.0089 (13)	0.0051 (13)	−0.0079 (12)
C32	0.0375 (19)	0.0330 (17)	0.0402 (19)	−0.0105 (15)	0.0101 (15)	−0.0167 (15)
C33	0.042 (2)	0.0367 (18)	0.0312 (17)	−0.0121 (16)	0.0003 (15)	−0.0164 (15)
C34	0.0264 (16)	0.0223 (15)	0.0301 (16)	−0.0086 (13)	0.0020 (13)	−0.0066 (12)
C35	0.0270 (18)	0.0326 (17)	0.047 (2)	−0.0073 (14)	0.0024 (15)	−0.0152 (15)
C36	0.0271 (17)	0.0320 (17)	0.0353 (17)	−0.0139 (14)	0.0002 (13)	−0.0130 (14)
O1W	0.108 (3)	0.0634 (19)	0.0524 (17)	−0.0390 (18)	−0.0031 (17)	−0.0143 (15)
O2W	0.0598 (17)	0.0577 (16)	0.0551 (16)	−0.0190 (14)	−0.0104 (13)	−0.0327 (13)
O3W	0.0644 (17)	0.0424 (14)	0.0417 (14)	−0.0231 (13)	0.0052 (12)	−0.0187 (11)

### Geometric parameters (Å, °)

**Table:**

Ni1—N11	1.956 (2)	N33—C34	1.394 (4)
Ni1—N21	1.960 (2)	N34—C36	1.339 (4)
Ni1—O13	2.067 (2)	N34—H34A	0.9184
Ni1—O23	2.110 (2)	N34—H34B	0.9241
Ni1—O11	2.121 (2)	S31—C32	1.711 (4)
Ni1—O21	2.164 (2)	S31—C33	1.736 (3)
Ni2—O4	2.041 (2)	S32—C35	1.720 (3)
Ni2—O1	2.056 (2)	S32—C36	1.740 (3)
Ni2—N31	2.067 (2)	C11—C12	1.375 (4)
Ni2—O3	2.0695 (19)	C11—C16	1.519 (4)
Ni2—N33	2.096 (2)	C12—C13	1.380 (4)
Ni2—O2	2.131 (2)	C12—H12	0.9300
O1—H1A	0.8609	C13—C14	1.390 (4)
O1—H1B	0.8248	C13—H13	0.9300
O2—H2A	0.7732	C14—C15	1.371 (4)
O2—H2B	0.7640	C14—H14	0.9300
O3—H3A	0.7774	C15—C17	1.523 (4)
O3—H3B	0.7478	C21—C22	1.381 (4)
O4—H4A	0.8275	C21—C26	1.515 (4)
O4—H4B	0.7402	C22—C23	1.386 (4)
O11—C16	1.267 (4)	C22—H22	0.9300
O12—C16	1.236 (3)	C23—C24	1.383 (4)
O13—C17	1.257 (3)	C23—H23	0.9300
O14—C17	1.235 (3)	C24—C25	1.373 (4)
O21—C26	1.276 (3)	C24—H24	0.9300
O22—C26	1.234 (3)	C25—C27	1.510 (4)
O23—C27	1.263 (4)	C31—C32	1.344 (4)
O24—C27	1.247 (4)	C31—C34	1.453 (4)
N11—C11	1.332 (4)	C32—H32	0.9300
N11—C15	1.337 (3)	C34—C35	1.340 (4)
N21—C21	1.330 (3)	C35—H35	0.9300
N21—C25	1.333 (4)	O1W—H1WA	0.9140
N31—C33	1.314 (4)	O1W—H1WB	0.7473
N31—C31	1.387 (4)	O2W—H2WB	0.7194
N32—C33	1.335 (4)	O2W—H2WA	0.7934
N32—H32A	0.9783	O3W—H3WA	0.7332
N32—H32B	0.9467	O3W—H3WB	0.8837
N33—C36	1.312 (4)		
			
N11—Ni1—N21	175.99 (9)	C35—S32—C36	89.37 (15)
N11—Ni1—O13	79.07 (9)	N11—C11—C12	120.2 (3)
N21—Ni1—O13	99.01 (9)	N11—C11—C16	112.4 (2)
N11—Ni1—O23	97.79 (9)	C12—C11—C16	127.4 (3)
N21—Ni1—O23	78.84 (9)	C11—C12—C13	119.0 (3)
O13—Ni1—O23	95.95 (9)	C11—C12—H12	120.5
N11—Ni1—O11	77.57 (8)	C13—C12—H12	120.5
N21—Ni1—O11	104.53 (9)	C12—C13—C14	119.6 (3)
O13—Ni1—O11	156.33 (8)	C12—C13—H13	120.2
O23—Ni1—O11	90.94 (9)	C14—C13—H13	120.2
N11—Ni1—O21	106.47 (9)	C15—C14—C13	118.9 (3)
N21—Ni1—O21	77.05 (8)	C15—C14—H14	120.6
O13—Ni1—O21	92.33 (8)	C13—C14—H14	120.6
O23—Ni1—O21	155.44 (8)	N11—C15—C14	120.2 (3)
O11—Ni1—O21	90.65 (8)	N11—C15—C17	111.9 (2)
O4—Ni2—O1	170.27 (9)	C14—C15—C17	127.9 (3)
O4—Ni2—N31	95.55 (9)	O12—C16—O11	126.0 (3)
O1—Ni2—N31	88.13 (9)	O12—C16—C11	119.3 (3)
O4—Ni2—O3	86.23 (8)	O11—C16—C11	114.7 (2)
O1—Ni2—O3	91.32 (8)	O14—C17—O13	125.6 (3)
N31—Ni2—O3	172.35 (9)	O14—C17—C15	119.2 (3)
O4—Ni2—N33	94.86 (9)	O13—C17—C15	115.2 (2)
O1—Ni2—N33	94.67 (9)	N21—C21—C22	120.8 (3)
N31—Ni2—N33	79.68 (9)	N21—C21—C26	113.1 (2)
O3—Ni2—N33	92.77 (9)	C22—C21—C26	126.1 (3)
O4—Ni2—O2	87.35 (9)	C21—C22—C23	118.2 (3)
O1—Ni2—O2	83.27 (8)	C21—C22—H22	120.9
N31—Ni2—O2	96.50 (9)	C23—C22—H22	120.9
O3—Ni2—O2	91.00 (8)	C24—C23—C22	120.1 (3)
N33—Ni2—O2	175.75 (8)	C24—C23—H23	119.9
Ni2—O1—H1A	116.2	C22—C23—H23	119.9
Ni2—O1—H1B	128.2	C25—C24—C23	118.5 (3)
H1A—O1—H1B	111.8	C25—C24—H24	120.7
Ni2—O2—H2A	119.6	C23—C24—H24	120.7
Ni2—O2—H2B	112.6	N21—C25—C24	120.9 (3)
H2A—O2—H2B	100.9	N21—C25—C27	112.8 (3)
Ni2—O3—H3A	115.4	C24—C25—C27	126.3 (3)
Ni2—O3—H3B	109.9	O22—C26—O21	126.0 (3)
H3A—O3—H3B	105.8	O22—C26—C21	119.1 (3)
Ni2—O4—H4A	132.5	O21—C26—C21	114.8 (2)
Ni2—O4—H4B	118.2	O24—C27—O23	125.7 (3)
H4A—O4—H4B	106.3	O24—C27—C25	118.3 (3)
C16—O11—Ni1	115.03 (18)	O23—C27—C25	116.1 (3)
C17—O13—Ni1	115.52 (18)	C32—C31—N31	115.6 (3)
C26—O21—Ni1	114.41 (18)	C32—C31—C34	128.2 (3)
C27—O23—Ni1	113.85 (19)	N31—C31—C34	116.2 (2)
C11—N11—C15	122.1 (2)	C31—C32—S31	110.3 (3)
C11—N11—Ni1	119.51 (19)	C31—C32—H32	124.8
C15—N11—Ni1	117.94 (19)	S31—C32—H32	124.8
C21—N21—C25	121.5 (2)	N31—C33—N32	124.3 (3)
C21—N21—Ni1	120.25 (19)	N31—C33—S31	113.6 (3)
C25—N21—Ni1	117.90 (19)	N32—C33—S31	122.0 (3)
C33—N31—C31	110.7 (3)	C35—C34—N33	115.4 (3)
C33—N31—Ni2	134.9 (2)	C35—C34—C31	129.0 (3)
C31—N31—Ni2	114.33 (19)	N33—C34—C31	115.5 (3)
C33—N32—H32A	114.5	C34—C35—S32	110.6 (2)
C33—N32—H32B	119.9	C34—C35—H35	124.7
H32A—N32—H32B	121.6	S32—C35—H35	124.7
C36—N33—C34	110.7 (3)	N33—C36—N34	125.1 (3)
C36—N33—Ni2	135.7 (2)	N33—C36—S32	113.9 (2)
C34—N33—Ni2	113.26 (19)	N34—C36—S32	121.0 (2)
C36—N34—H34A	111.4	H1WA—O1W—H1WB	107.0
C36—N34—H34B	116.1	H2WB—O2W—H2WA	104.5
H34A—N34—H34B	126.2	H3WA—O3W—H3WB	107.6
C32—S31—C33	89.77 (16)		

### Hydrogen-bond geometry (Å, °)

**Table:**

*D*—H···*A*	*D*—H	H···*A*	*D*···*A*	*D*—H···*A*
O1—H1A···O14^i^	0.86	1.75	2.606 (3)	174
O1—H1B···O21^ii^	0.82	1.92	2.747 (3)	176
O2—H2A···O12^iii^	0.77	2.01	2.772 (3)	169
O2—H2B···O22^ii^	0.76	2.12	2.874 (3)	170
O3—H3A···O3W^ii^	0.78	1.98	2.742 (3)	169
O3—H3B···O11^iii^	0.75	1.94	2.683 (3)	173
O4—H4A···O24^iv^	0.83	1.89	2.717 (4)	179
O4—H4B···O1W	0.74	2.16	2.820 (4)	149
O1W—H1WA···O23^iv^	0.91	2.01	2.907 (4)	166
O1W—H1WB···O24^iii^	0.75	2.41	3.150 (5)	169
O2W—H2WB···O3W^v^	0.72	2.41	3.104 (4)	162
O2W—H2WA···O14	0.79	2.02	2.815 (4)	174
O3W—H3WA···O22^vi^	0.73	2.24	2.934 (3)	158
O3W—H3WB···O21	0.88	2.10	2.892 (3)	149
N32—H32A···O12	0.98	1.97	2.940 (5)	169
N32—H32B···O2	0.95	2.19	2.996 (5)	142
N34—H34A···O2W^vii^	0.92	1.98	2.886 (4)	168
N34—H34B···O3	0.92	2.12	2.952 (4)	149
C12—H12···S31	0.93	2.71	3.631 (4)	170

Symmetry codes: (i) −*x*+1, −*y*, −*z*+1; (ii) *x*, *y*, *z*+1; (iii) −*x*, −*y*+1, −*z*+1; (iv) *x*, *y*+1, *z*; (v) −*x*+1, −*y*, −*z*; (vi) −*x*, −*y*+1, −*z*; (vii) *x*, *y*+1, *z*+1.
